# Transapical Transcatheter Aortic Valve Replacement Under 3-Dimensional Guidance to Treat Pure Aortic Regurgitation in Patients with a Large Aortic Annulus

**DOI:** 10.31083/j.rcm2509319

**Published:** 2024-09-09

**Authors:** Yu Mao, Yang Liu, Mengen Zhai, Ping Jin, Lai Wei, Haibo Zhang, Jian Liu, Xiangbin Pan, Yingqiang Guo, Jian Yang

**Affiliations:** ^1^Department of Cardiovascular Surgery, Xijing Hospital, 710032 Xi’an, Shaanxi, China; ^2^Department of Cardiovascular Surgery, Shanghai Cardiovascular Institution and Zhongshan Hospital, Fudan University, 200433 Shanghai, China; ^3^Department of Cardiovascular Surgery, Anzhen Hospital, Capital Medical University, 100069 Beijing, China; ^4^Department of Cardiovascular Surgery, Guangdong Provincial People’s Hospital, 519041 Guangzhou, Guangdong, China; ^5^Department of Cardiology, Fuwai Hospital, National Center for Cardiovascular Disease, Chinese Academy of Medical Science and Peking Union Medical College, 100730 Beijing, China; ^6^Department of Cardiovascular Surgery, West China Hospital, Sichuan University, 610065 Chengdu, Sichuan, China

**Keywords:** aortic regurgitation, transcatheter aortic valve replacement, large annulus, 3-dimensional printing, extra oversizing

## Abstract

**Background:**

Transcatheter aortic valve replacement (TAVR) is a challenge for patients with aortic regurgitation (AR) and a large annulus. Our goal was to evaluate the clinical outcomes and predictors of transapical TAVR in AR patients with a large annulus and noncalcification and the feasibility and safety of 3-dimensional printing (3DP) in the preprocedural simulation.

**Methods:**

Patients with a large annulus (diameter >29 mm) were enrolled and divided into the simulation (n = 43) and the nonsimulation group (n = 82). Surgeons used the specific 3DP model of the simulation group to simulate the main steps before the procedure and to refit the transcatheter heart valve (THV) according to the simulated results.

**Results:**

The average annular diameter of the overall cohort was 29.8 ± 0.7 mm. Compared with the nonsimulation group, the simulation group used a higher proportion of extra oversizing for THVs (97.6% vs. 85.4%, *p* = 0.013), and the coaxiality performance was better (9.7 ± 3.9° vs. 12.7 ± 3.8°, *p* < 0.001). Both THV displacement and ≥ mild paravalvular leakage (PVL) occurred only in the nonsimulation group (9.8% vs. 0, *p* < 0.001; 9.8% vs. 0, *p* < 0.001). Multivariate regression analysis showed that extra oversizing, coaxial angle and annulus diameter were independent predictors of THV displacement and ≥ mild PVL, respectively.

**Conclusions:**

Based on 3DP guidance, transapical TAVR using extra oversizing was safe and feasible for patients with noncalcified AR with a large annulus. Extra oversizing and coaxial angle were predictors of postprocedural THV displacement and ≥ mild PVL in such patients.

## 1. Introduction

Aortic regurgitation (AR) is a common aortic valve (AV) disease. In China, epidemiological analysis based on echocardiography suggests that the incidence of ≥moderate AR in patients ≥75 years old was 2.9%, and the proportion of patients with severe AR is significantly higher than that of patients with severe aortic stenosis (AS) [1]. Furthermore, the study confirms that the natural prognosis of chronic severe AR in patients with symptoms is extremely poor, with the annual fatality rate reaching 24.6% in patients with New York Heart Association (NYHA) functional class III/IV [2]. At present, surgical AV replacement (SAVR) is still preferred for most AR patients with intervention indications [3, 4]. However, only 20.0% of patients with pure AR with a left ventricular ejection fraction (LVEF) of 30%–50% undergo SAVR. Notably, when LVEF is less than 30%, the proportion of such patients is as low as 3% [5].

Transcatheter AV replacement (TAVR) has developed rapidly in recent years, and breakthroughs have been made in the treatment of high-risk patients with AR [6]. However, its success rate is lower than that for patients with AS [7]. The main challenges lie in the lack of calcification in the annulus [8, 9], the relatively large and elliptical annulus [10], the pathologically dilated aortic root and ascending aorta [11, 12], and the poorer cardiac function [10]. The J-Valve prosthesis (Jiecheng Medical Co., LTD., Suzhou, China) used in this study has proven to be safe and effective in clinical studies due to its unique positioning key design [13]. However, it should be noted that the maximum size of the transcatheter heart valve (THV) is 29 mm and that TAVR performed in patients with AR with a larger annulus is often encountered and thus has become a hot research topic. Due to the large annulus in such patients, the THV anchoring is more difficult than in patients with normal size annulus. Meanwhile, the shear stress after implantation is weak, thus THV malposition caneasily occur. Additionally, such patients are often accompanied by dilation of the ascending aorta and aortic sinuses, which undoubtedly adds to the difficulty of procedures. Therefore, for patients with noncalcified AR with a large annulus, the existing THV is still challenging to match [14, 15].

Traditionally, computed tomography angiography (CTA) is sometimes limited in its ability to properly display the THV performance after implantation [16, 17]. The advent of cardiovascular 3-dimensional printing (3DP) may be an innovative solution to this challenging situation, with previous studies confirming the fact that preprocedural planning enabled by the technology may significantly improve anatomical visualization, provide comprehensive THV sizing recommendations, and help determine the THV implanted position and identify any potential complications through preprocedural simulations [18, 19, 20]. Therefore, our goal was to evaluate the clinical outcomes and predictors of transapical TAVR in patients with AR with a large annulus and noncalcification, as well as the feasibility and safety of 3D printing in a preprocedural simulation.

## 2. Material and Methods

### 2.1 Study Design and Population

This prospective study included 125 patients with noncalcified AR with a large annulus (annular diameter >29 mm) who underwent transapical TAVR at 9 high-volume centers from January 2018 to May 2021. Inclusion criteria included age ≥60 years, NYHA functional class ≥II, ≥moderate AR diagnosed by transthoracic echocardiography (TTE); European System for Cardiac Operative Risk Evaluation score II >12% or Society of Thoracic Surgeons (STS) score >8%. The exclusion criteria were <moderate AR; a myocardial infarction that occurred within 1 month; a history of endocarditis; hypertrophic cardiomyopathy; and a transient ischemic attack/stroke that occurred within 6 months. In addition, patients unsuitable for transapical TAVR were excluded from the study. The 3D printing simulation was performed alternately in each subset of 3 consecutive patients per center (One patient was assigned to the simulation group and the other two patients were assigned to the nonsimulation group). Therefore, all 125 patients were divided into the simulation group (n = 43) and the nonsimulation group (n = 82). This study complied with the Declaration of Helsinki and was approved by the local ethics commissions. All patients provided written informed consent for the procedures and for subsequent data collection.

### 2.2 Preoperative Imaging Assessment

All patients underwent CTA and TTE before having the procedure. The CTA scans were collected by the retrospective electrocardiogating method and then were imported into Materialise Mimics Version 21.0 (Materialise, Leuven, Belgium) for pre-TAVR evaluations. Preprocedural evaluation mainly included the diameters of the aortic annulus, the aortic sinuses, the left ventricular outflow tract, the coronary artery heights, and the optimal projection angles. Transthoracic echocardiography mainly assesses the degree of regurgitation, pressure gradient, LVEF, and other cardiac complications.

### 2.3 3-Dimensional Printing Simulation

**Fig. 1.**
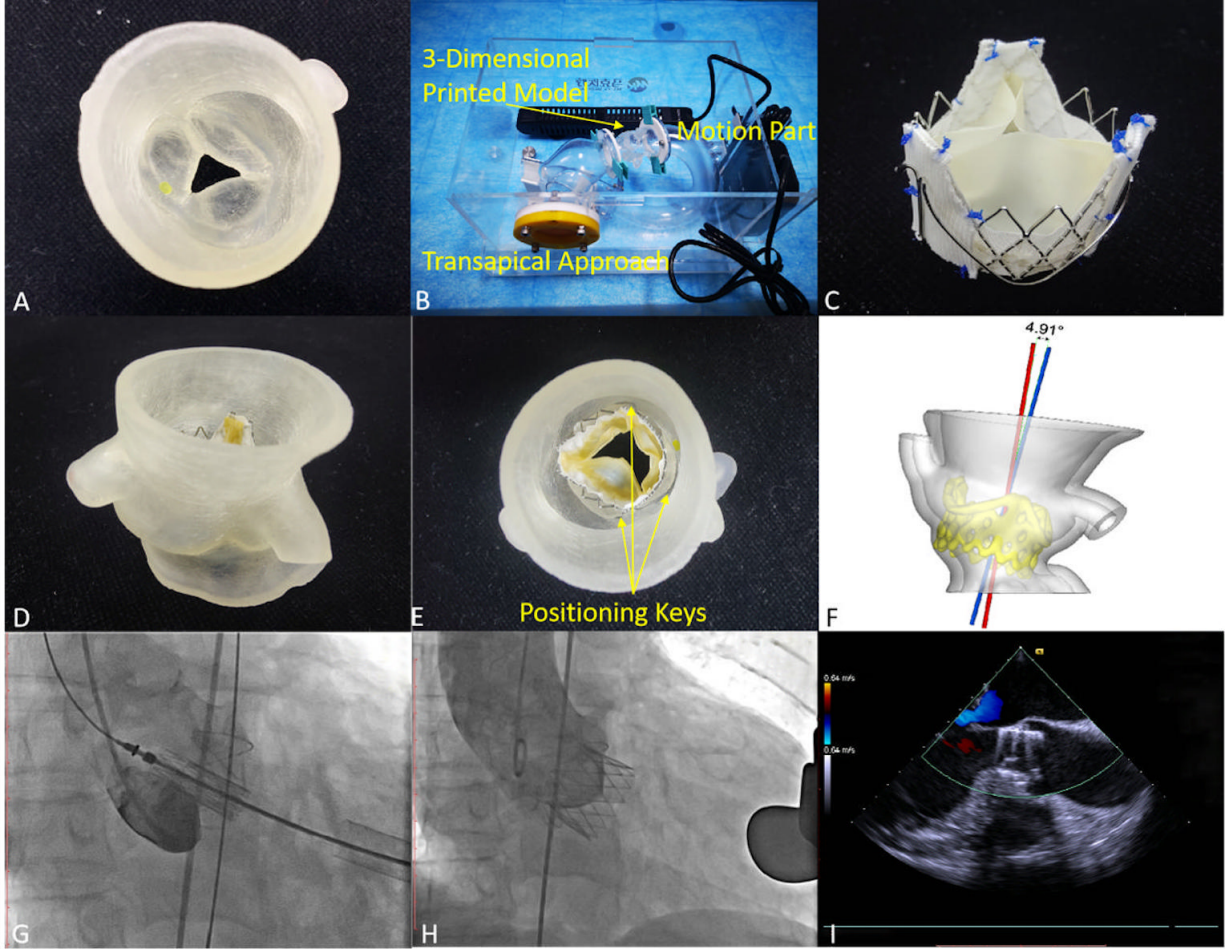
**Preoperative simulation of transapical transcatheter aortic valve replacement with additional dimensions using 3-dimensional printed models**. (A) The 3-dimensional printed model from the ascending aorta plane. (B) The pulsatile simulator using procedural simulations. The arrows pointed to the components of the simulator. (C) A polyester patch was stitched onto the prosthesis. After a simulated implant, the transcatheter heart valve displacement (D) and the occurrence of ≥ mild paravalvular leakage (E) could be observed clearly in the model. The arrows pointed to the positioning keys. (F) The coaxial angle was measured after the simulations. During the procedures, the device was positioned (G) and then successfully unfolded (H,I), and the immediate transesophageal echocardiography after procedures verified the procedural effect.

*3-Dimensional printed model*. After the scans were collected, the reconstructed 3D model of the aortic root was obtained using Materialise Mimics Version 21.0 (Leuven, Belgium) software. Then, the aortic root model was completely restored using Materialise 3-matic (Leuven, Belgium) software and imported into the Stratasys Polyjet 850 multimaterial full color 3D printer, and different materials were selected according to different tissues for matching (Fig. 1A).

*Pulsating simulator*. The simulator is composed of two parts: The working part includes the aortic approach, the specific 3D printed model, and the approach for transesophageal echocardiography (TEE). The power part is composed of the circulation pump, the circulating connecting loop, and the control system (Fig. 1B).

*Extra Oversizing*. Before the THV was preassembled into the delivery system, a Dacron patch was cut to the size corresponding to the three positioning keys. Then, 6 to 8 stitches were made between the Dacron patch and the three positioning keys with 6-0 Prolene suture to widen the THV diameter by 3–4 mm and increase its friction (Fig. 1C). After the simulations were made for the THV, whose diameter was not sufficient to stabilize the annulus, the surgeons inserted another 6-0 Prolene suture at the lower margin of the THV to assist in anchoring. Finally, the refitted THV was soaked in ice water and then ballasted into the delivery system.

*3-Dimensional printing simulation*. After the specific 3D printed aortic root model in the simulation group was obtained, the model was assembled into the pulsating simulator. Through several simulations, surgeons could determine whether to use the THV extra oversizing to improve the accuracy of the implant depth and the coaxiality. In particular, we paid attention to the content after the THV was released: (i) THV displacement (Fig. 1D); (ii) paravalvular leakage (PVL) (Fig. 1E); (iii) coaxial angle (after the simulations, the model with the implanted prosthesis was scanned by micro-computed tomography to measure the angle between the central axis of the aortic root and the central axis of the THV) (Fig. 1F).

### 2.4 Procedures

The patient was given general anesthesia and then intubated. The internal jugular vein catheter was implanted in the right side of the neck, and the atrial pacing sheath was then implanted and tested to see whether it worked normally. After routine heparinization was performed, a 4- to- 6-cm surgical incision was made between the fifth and sixth intercostal spaces lateral to the left midclavicular line. After fully exposing the apex cordis, the pericardium was incised and suspended, and a round pouch with a diameter of 1 cm was prefabricated with 3-0 Prolene suture and a felt sheet. A 6 Fr sheath catheter was implanted from the left femoral artery, then a pigtail catheter was implanted into the bottom of the right-/noncoronary sinus for intraprocedural positioning. Digital subtraction angiography (DSA) identified the annulus and the coronary arteries.

The surgeons decided to use the THV extra oversizing before performing the procedure based on the evaluations of each specific patient (extra oversizing steps as described previously). Subsequently, the position for the transapical TAVR was found using DSA guidance, and a 16- to 20-Fr vascular sheath was inserted through a transapical puncture. The 5 Fr multifunctional angiographic catheter (Johnson & Johnson, New Brunswick, NJ, USA) was placed anteriorly across the AV under the guidance of a 150-cm super-loach guide wire to reach the horizontal line of the abdominal aorta, and a 260-cm Lunderquist guide wire was exchanged. The delivery system was inserted into the AV along a rigid guide wire, and the J-Valve delivery system was inserted through the transapical approach. The positioning keys were opened to enter the sinuses (Fig. 1G). The DSA and TEE were used to determine that the positioning keys were positioned in the sinuses. Then, with the support of the guide wire, the THV was released. Both DSA and TEE were performed to evaluate the position and function of the THV immediately (Fig. 1H,I). After the confirmation, the guide wire and delivery system were removed, and the pouch of the apex cordis was sutured. Finally, DSA again confirmed that the coronary artery opening was not blocked, the THV was positioned correctly, and the leaflets opened and closed normally.

### 2.5 End Points

According to the Valvular Academic Research Consortium-3 criteria [21], the primary end point of the study was the device success. Secondary end points included 2-year all-cause mortality, incidence of related complications (newly implanted permanent pacemaker and other procedural- and device-related complications), and TTE assessment of THV and cardiac function.

### 2.6 Statistical Analyses

All data were tested for normality and homogeneity of variance. Continuous variables were expressed as mean ± standard deviation or median and quartile ranges. The results of the classified data were expressed as n (%). The Student *t*-test and Mann-Whitney U test were used to compare normally distributed continuous and continuously non-normally distributed variables, respectively, using the Chi-square test or the Fisher exact test when appropriate. Univariate and multivariate logistic regression analyses were performed for possible correlation factors. Multivariate models included statistically significant variables with a univariate analysis *p*-value < 0.05. Survival curves for all-cause mortality were constructed using Kaplan-Meier estimates and compared using log-rank statistics. Bilateral *p*-values < 0.05 were considered statistically significant. All statistical analyses were performed using the R programming language version 4.2.2 (R Foundation for Statistical Computing, Vienna, Austria).

## 3. Results

### 3.1 Baseline Characteristics

Baseline characteristics of the overall cohort are shown in Table 1. The average age was 72.5 ± 5.6 years; 70.4% (n = 88) were males; and the STS score was 8.3 ± 3.5%. There was no significant difference in the proportion of NYHA functional class ≥III between the two groups. In addition, there was no significant difference in the incidence of the patient’s previous medical history, except that the simulation group had a lower incidence of chronic obstructive pulmonary disease than the nonsimulation group (2.3% vs. 12.2%, *p* = 0.064).

**Table 1.** **Baseline characteristics of patients who underwent transcatheter aortic valve replacement**.

Characteristics		Overall cohort (n = 125)	3DP simulation group (n = 43)	Non-3DP simulation group (n = 82)	*p*-value
Demographics					
	Age, mean (SD), years	72.5 (5.6)	73.2 (5.0)	71.6 (5.8)	0.313
	Men, %	88 (70.4)	31 (72.1)	57 (69.5)	0.764
	Body mass index, mean (SD), kg/m^2^	22.8 (2.0)	22.5 (2.0)	23.0 (2.0)	0.159
	NYHA functional class ≥III, %	123 (98.4)	43 (100)	80 (97.6)	0.545
	STS score, mean (SD), %	8.3 (3.5)	8.4 (3.0)	8.3 (3.4)	0.909
Comorbidities					
	Diabetes, %	24 (19.2)	10 (23.3)	14 (17.1)	0.405
	Hypertension, %	29 (23.2)	9 (20.9)	20 (24.4)	0.663
	Peripheral vascular disease, %	68 (54.4)	24 (55.8)	44 (53.7)	0.818
	Stroke/transient ischemic attack, %	3 (2.4)	1 (2.3)	2 (2.4)	1.000
	Coronary artery disease, %	29 (23.2)	8 (18.6)	21 (25.6)	0.378
	Previous percutaneous coronary intervention, %	5 (4.0)	2 (4.7)	3 (3.7)	1.000
	Previous bypass graft surgery, %	29 (23.2)	8 (18.6)	21 (25.6)	0.378
	Chronic obstructive pulmonary disease, %	11 (8.8)	1 (2.3)	10 (12.2)	0.064
	Dialysis, %	29 (23.2)	9 (20.9)	20 (24.4)	0.663
	Atrial fibrillation, %	29 (23.2)	8 (18.6)	21 (25.6)	0.378
	Permanent pacemaker, %	4 (3.2)	3 (7.0)	1 (1.2)	0.229
	Bicuspid aortic valve, %	8 (6.4)	3 (7.0)	5 (6.1)	0.849

3DP, three-dimensional printing; NYHA, New York Heart Association; SD, standard deviation; STS, Society of Thoracic Surgeons.

The results of preprocedural CTA and TTE measurements are summarized in Table 2. The average annular diameter was 29.9 ± 0.7 mm, and the diameter of the left ventricular outflow tract was 31.9 ± 1.5 mm. Furthermore, there was no significant difference in preprocedural imaging measurements.

**Table 2.** **Preoperative assessment of patients who underwent transcatheter aortic valve replacement**.

Characteristics		Overall cohort (n = 125)	3DP simulation group (n = 43)	Non-3DP simulation group (n = 82)	*p*-value
Preoperative computed tomography angiography					
	Annulus diameter, mean (SD), mm	29.9 (0.8)	29.9 (0.7)	29.8 (0.8)	0.490
	LVOT diameter, mean (SD), mm	31.9 (1.5)	32.1 (1.9)	31.9 (1.2)	0.473
	STJ diameter, mean (SD), mm	40.5 (2.1)	40.6 (2.5)	40.5 (1.9)	0.803
	AA diameter, mean (SD), mm	43.1 (2.4)	43.1 (2.7)	43.0 (2.3)	0.828
	LCH, mean (SD), mm	15.4 (3.4)	15.2 (3.8)	15.6 (3.1)	0.528
	RCH, mean (SD), mm	19.7 (3.2)	19.4 (3.7)	19.9 (2.8)	0.399
	Aorta angle, mean (SD), °	54.8 (9.0)	54.8 (9.0)	54.8 (9.1)	1.000
	LVLD, mean (SD), mm	88.6 (10.1)	88.1 (11.2)	88.8 (9.5)	0.714
	LVAPD, mean (SD), mm	68.4 (8.6)	68.1 (8.8)	68.5 (8.5)	0.805
	LVLRD, mean (SD), mm	68.2 (8.0)	67.8 (9.6)	68.4 (8.0)	0.711
Preoperative transthoracic echocardiography					
	AV V_max_, mean (SD), cm/s	171.3 (23.7)	166.9 (24.6)	173.6 (23.1)	0.134
	MTVPG, mean (SD), mmHg	7.4 (3.2)	6.7 (2.8)	7.8 (3.3)	0.065
	Severe aortic regurgitation, %	101 (80.8)	34 (79.1)	67 (81.7)	0.722
	LVEF, mean (SD), %	49.9 (7.6)	49.2 (7.3)	50.3 (7.8)	0.446
	LVFS, mean (SD), %	25.9 (4.7)	25.1 (4.3)	26.3 (4.8)	0.172

AA, ascending aorta; AV V_max_, peak flow velocity of aortic valve; LCH, left coronary artery height; LVAPD, left ventricular anteroposterior diameter; LVEF, left ventricle ejection fraction; LVFS, left ventricular fraction shortening; LVLD, left ventricular long diameter; LVLRD, left ventricular left-right diameter; LVOT, left ventricular outflow tract; MTVPG, mean transvalvular pressure gradient; RCH, right coronary artery height; SD, standard deviation; STJ, sinotubular junction; 3DP, three-dimensional printing.

### 3.2 Procedural and Hospitalization Characteristics

**Fig. 2.**
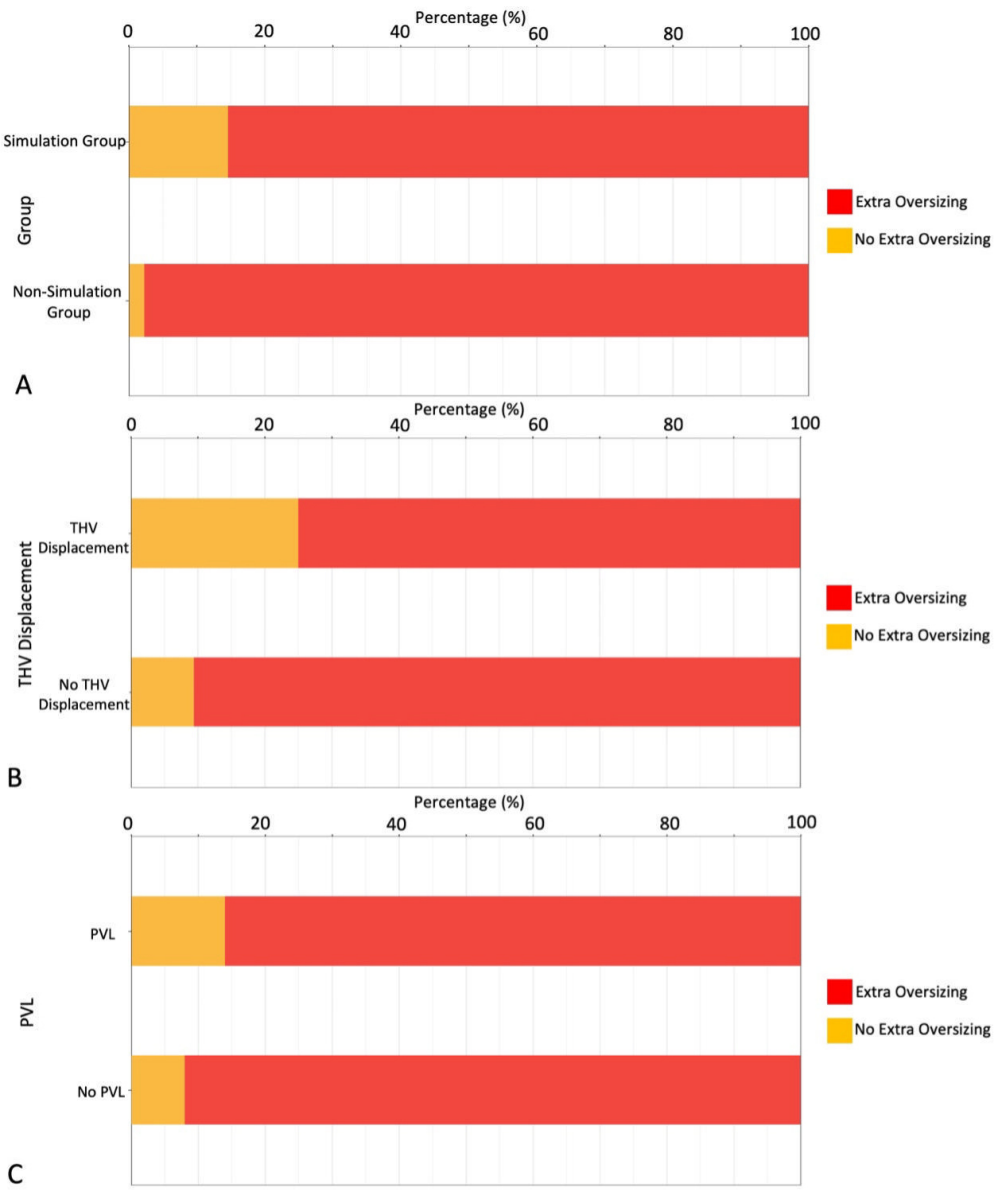
**Procedural extra oversizing outcomes**. (A) Comparison of extra oversizing between the two groups. (B,C) Correlations of extra oversizing with the incidence of transcatheter heart valve displacement and ≥ mild paravalvular leakage. THV, transcatheter heart valve; PVL, paravalvular leakage.

The procedural and in-hospital details are shown in Table 3. THV Displacement and ≥ mild PVL occurred only in the nonsimulation group (9.8% vs. 0, *p* = 0.05; 9.8% vs. 0, *p* = 0.05). Based on the preprocedural 3D printing simulations, the proportion of the THV with extra oversizing was higher than that of the nonsimulation group (97.6% vs. 86.0%, *p* = 0.013) (Fig. 2A). In the analysis of THV displacement, the proportion of extra oversizing in the no THV displacement population was larger than that in the THV displacement population (90.6% vs. 75.0%). Correspondingly, similar results were obtained in the analysis of PVL occurrence (92.2% vs. 88.9%) (Fig. 2B,C). The total operation time, DSA time, and contrast volume in the nonsimulation group were higher than those in the simulation group (118.5 ± 15.6 min vs. 104.8 vs. 15.7 min, *p* < 0.001; 9.2 ± 2.7 min vs. 6.1 vs. 2.3 min, *p* < 0.001; 731.7 ± 123.7 mGy vs. 518.8 vs. 57.7 mGy, *p* < 0.001) (**Supplementary Fig. 1**). In addition, in the nonsimulation group, 6 patients converted to SAVR, 2 patients had device embolization, 1 patient had a coronary artery obstruction, and 1 patient had a second valve implanted.

**Table 3.** **Procedural and hospitalization outcomes of patients who underwent transcatheter aortic valve replacement**.

Characteristic		Overall cohort (n = 125)	3DP simulation group (n = 43)	Non-3DP simulation group (n = 82)	*p*-value
Intraprocedural outcomes					
	Extra oversizing, %	117 (93.6)	37 (86.0)	80 (97.6)	0.012
	Operation time, mean (SD), min	113.8 (16.9)	104.8 (15.7)	118.5 (15.6)	<0.001
	DSA time, mean (SD), min	8.1 (3.0)	6.1 (2.3)	9.2 (2.7)	<0.001
	Radiation amount, mean (SD), mL	658.5 (146.4)	518.8 (57.7)	731.7 (123.7)	<0.001
Postprocedural outcomes					
	Conversion to open heart surgery, %	6 (4.8)	0 (0)	6 (7.3)	0.093
	Device displacement, %	8 (6.4)	0 (0)	8 (9.8)	0.050
	Tamponade, %	2 (1.6)	0 (0)	2 (2.4)	0.545
	Coronary obstruction, %	1 (0.8)	0 (0)	1 (1.2)	1.000
	Valve-in-valve implant, %	1 (0.8)	0 (0)	1 (1.2)	1.000
Paravalvular leakage					
	Mild, %	8 (6.4)	0 (0)	8 (9.8)	0.050
	Moderate, %	1 (0.8)	0 (0)	1 (1.2)	1.000
Implanted depth					
	LCC depth, mean (SD), mm	10.3 (2.9)	10.0 (2.7)	10.4 (3.1)	0.435
	RCC depth, mean (SD), mm	7.9 (2.8)	7.9 (2.7)	7.9 (2.9)	0.945
	NCC depth, mean (SD), mm	7.7 (2.9)	7.8 (2.8)	7.7 (3.0)	0.655
Hospitalization outcomes					
	Death, %	2 (1.6)	0 (0)	2 (2.4)	0.545
	Major adverse cardiovascular events, %	2 (1.6)	0 (0)	2 (2.4)	0.545
	Stroke, %	0 (0)	0 (0)	0 (0)	1.000
	Bleeding (major or life-threatening), %	0 (0)	0 (0)	0 (0)	1.000
	Major vascular complications, %	1 (0.8)	0 (0)	1 (1.2)	1.000
	Third-degree atrioventricular block, %	4 (3.2)	0 (0)	4 (4.9)	0.298
	Acute kidney injury (stage 3), %	2 (1.6)	0 (0)	2 (2.4)	0.545
	Coaxiality angle, mean (SD), °	11.3 (3.8)	9.7 (3.9)	12.7 (3.8)	<0.001
	ICU, mean (SD), days	1.6 (0.8)	1.3 (0.6)	1.8 (0.8)	<0.001
	Hospitalization, mean (SD), days	6.6 (3.2)	9.5 (3.5)	8.5 (3.6)	0.139

3DP, 3-dimensional printing; DSA, digital subtraction angiography; ICU, intensive care unit; LCC, left coronary cusp; NCC, noncoronary cusp; RCC, right coronary cusp; SD, standard deviation.

**Fig. 3.**
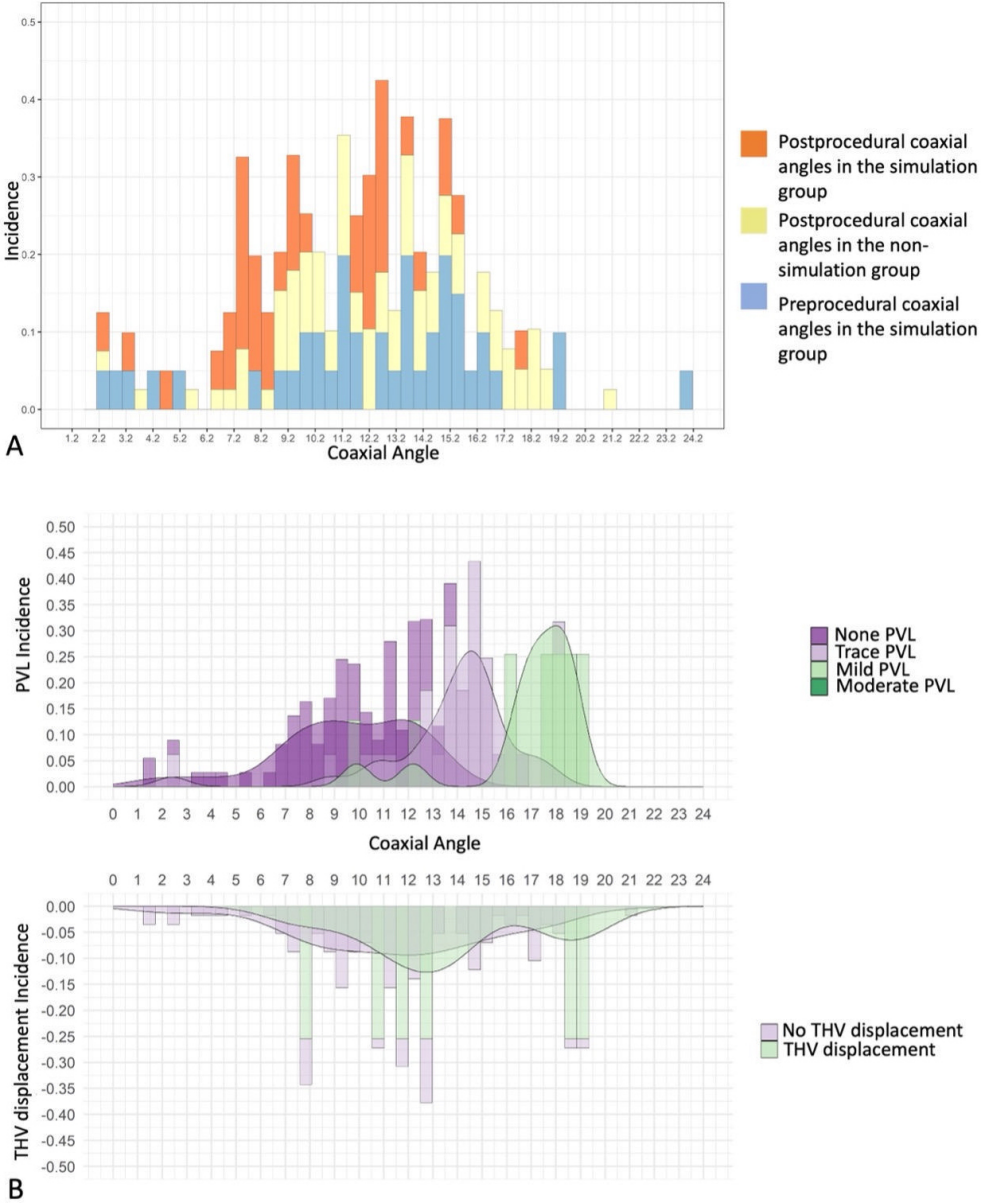
**Coaxiality angle performance**. (A) Comparison of coaxial angle measured by the simulation group before the procedure; the simulation group and the nonsimulation group after the procedure. (B) Correlation of the coaxial angle with the transcatheter heart valve displacement and ≥ mild paravalvular leakage. THV, transcatheter heart valve; PVL, paravalvular leakage.

No deaths or major complications occurred during hospitalization in the simulation group. Two deaths occurred in the nonsimulation group (two patients died of myocardial infarction and malignant arrhythmia, respectively). Based on the simulations, the coaxial angle in the simulation group was smaller than that in the nonsimulation group (9.7 ± 3.9° vs. 12.7 ± 3.8°, *p* < 0.001) (Fig. 3A). In addition, 4 patients in the nonsimulation group developed third degree atrioventricular block after undergoing the procedure.

### 3.3 Clinical Outcomes

**Fig. 4.**
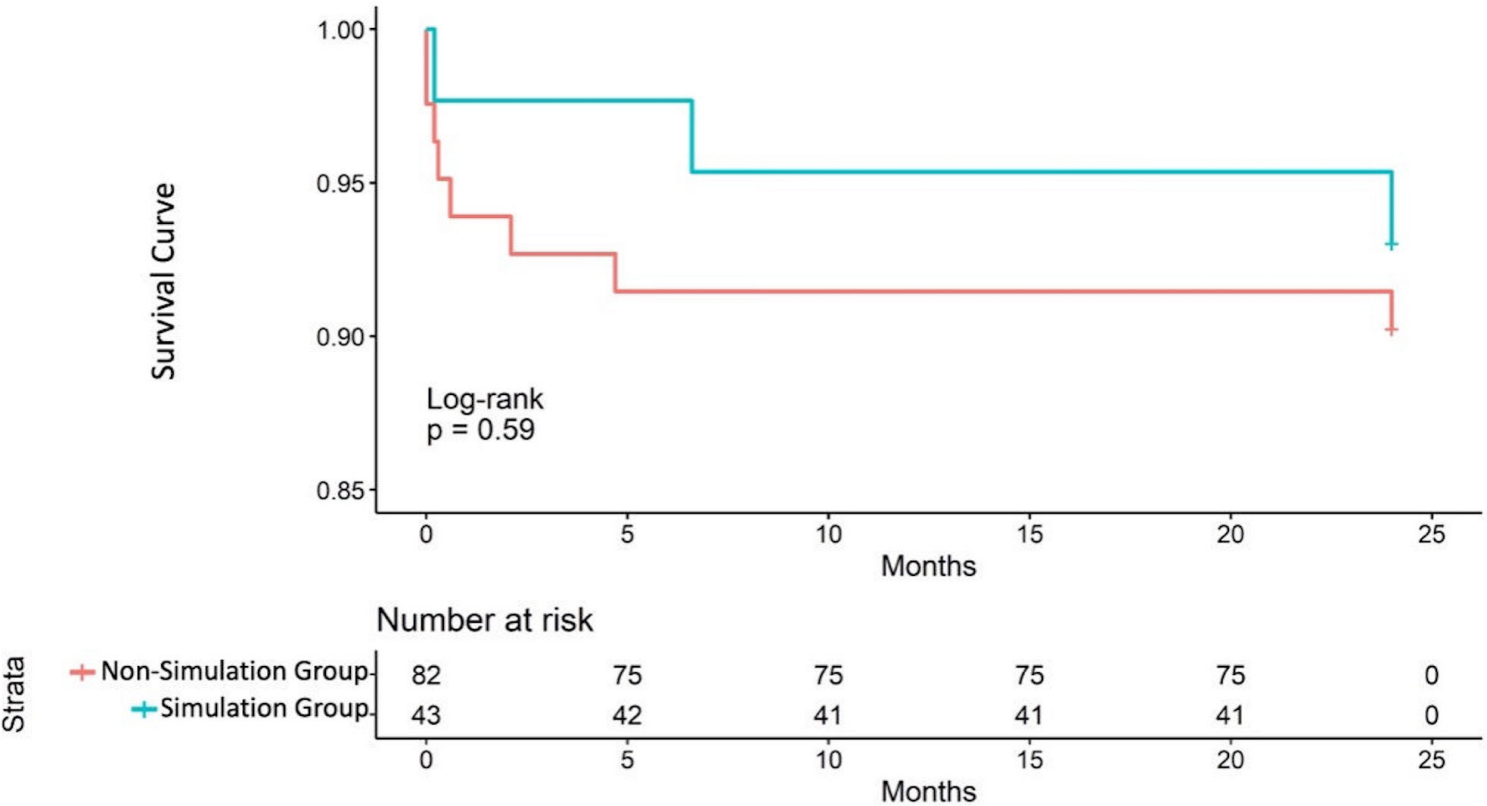
**Two-year survival curves of the simulation and nonsimulation groups**.

Multivariate regression analysis showed that the larger annular diameter [odds ratio (OR): 1.03; 95% confidence interval (CI): 1.07–1.73; *p* = 0.004] and the aortic angulation (OR: 1.10, 95% CI: 1.04–1.78; *p* = 0.003) were independent risk factors for THV displacement. Meanwhile, the larger annular diameter (OR: 1.18, 95% CI: 1.07–1.37; *p* < 0.001) and bicuspid AV morphology (OR: 2.49, 95% CI: 1.23–4.60; *p* < 0.001) were risk factors that increased the incidence of ≥ mild PVL. However, extra oversizing was the protective factor that prevented THV displacement (OR: 0.61, 95% CI: 0.43–0.81; *p* < 0.001) and ≥ mild PVL (OR: 0.66, 95% CI: 0.25–0.90; *p* < 0.001) (Table 4). Interestingly, the coaxial angle was the independent protective factor of THV displacement (OR: 0.68, 95% CI: 0.32–0.89; *p* < 0.001) and ≥ mild PVL (OR: 0.58, 95% CI: 0.21–0.72; *p* < 0.001) (Table 4, Fig. 3B). The Kaplan-Meier survival curve for 2-year all-cause mortality is shown in Fig. 4. At the 2-year follow-up, the overall survival rate was 92.8%. Among them, there were 2 deaths in the simulation group and 7 deaths in the nonsimulation group.

**Table 4.** **Logistic regression analysis of transcatheter heart valve displacement and paravalvular leakage**.

Characteristics		Univariate analysis			Multivariate analysis		
		Odds ratio	95% CI	*p-*value	Odds ratio	95% CI	*p-*value
Predictors of THV displacement							
	Atrial fibrillation	1.12	0.33–3.78	0.86			
	MTVPG	1.17	0.23–2.56	0.67			
	LVFS	1.06	0.72–2.79	0.76			
	Annulus diameter	1.04	1.19–2.25	<0.001	1.03	1.07–1.73	0.004
	Horizocardia	1.67	1.20–2.31	<0.001	1.10	1.04–1.78	0.003
	Coaxial angle	0.53	0.16–0.84	<0.001	0.68	0.32–0.89	<0.001
	Extra oversizing	0.51	0.11–0.70	<0.001	0.61	0.43–0.81	<0.001
Predictors of PVL grade ≥ mild							
	Body mass index	1.20	0.21–2.85	0.84			
	Bicuspid aortic valve	3.42	1.43–6.06	<0.001	2.49	1.23–4.60	<0.001
	AV V_max_	1.65	0.70–2.08	0.69			
	Annulus diameter	1.42	1.34–1.63	<0.001	1.18	1.07–1.37	<0.001
	Horizocardia	0.94	0.85–1.03	0.38			
	Coaxial angle	0.45	0.08–0.63	<0.001	0.58	0.21–0.72	<0.001
	Extra oversizing	0.49	0.14–0.81	<0.001	0.66	0.25–0.90	<0.001

AV V_max_, peak flow velocity of aortic valve; CI, confidence interval; LVFS, left ventricular fraction shortening; MTVPG, mean transvalvular pressure gradient; PVL, paravalvular leakage; THV, transcatheter heart valve.

## 4. Discussion

The purpose of this study was to evaluate the clinical efficacy and predictors of transapical TAVR in patients with noncalcified AR with a large annulus and the feasibility and safety of 3D printing in preprocedural simulation. The main findings were (i) TAVR performed using the J-Valve and the extra oversizing technique was safe and feasible for such patients; (ii) Extra oversizing and the coaxial angle were predictors of THV displacement and ≥ mild PVL; (iii) After the preprocedural simulations, the surgeons effectively reduced the incidence of THV displacement and ≥ mild PVL by increasing the usage rate of extra oversizing and adjusting the coaxial angle.

The study results of the American Association of Thoracic Surgeons and the American College of Cardiology showed that the number of TAVRs performed in 2019 exceeded the number of SAVRs [22]. Almost all patients treated in the registry had AS. At present, patients with AR are still treated mainly with SAVR [3, 4]. Researchers [23] also began to try interventional treatment for patients with AR, and relevant study results confirmed that TAVR was feasible for such patients.

However, few clinical studies on patients with AR with a large annulus have been published. One reason may be that the large annulus in such patients makes it difficult to implant the THV in the aortic root. Secondly, the shear force of the implanted THV decreases due to the large annulus, which may cause the THV to immediately fall off and shift to the ascending aorta or aortic arch.

In this study, the J-Valve system was designed with three U-shaped positioning keys that fit the anatomical structures of the aortic sinuses. The THV requires a two-step release for more accurate positioning and leaflet clamping [23]. However, for patients with a large annulus, a valve implanted in the noncalcified AV area is prone to shift, increasing the risk of THV displacement. Patients with AR also have relative pathological dilation in the aortic root and ascending aorta, resulting in further annular enlargement [24]. Because the annulus of such patients exceeds the maximum available size of the J-Valve, the force of the clamp between the device and the leaflets might be weakened, leading to an increased risk of it falling off after the THV is implanted. Thus, the adjustment of THV size and coaxiality and the stable positioning and anchoring in the annulus are technical challenges for TAVR in such patients. Studies of similar products showed that downsizing or oversizing the CoreValve and the Evolut R easily led to poor coaxiality of the implanted THV. The preceding factors may be related to the increased risk of incorrect positioning [25, 26]. However, the preliminary results of the JenaValve system suggest that the transfemoral TAVR system may have some advantages in avoiding aforementioned problems [27]. In this study, for patients with a >29-mm annulus predicted by preprocedural imaging assessments, the Dacron patches were sutured to the margins of three positioning keys to increase the friction with the aortic root, thereby reducing the risk of THV displacement after implantation (Comparsion of the extra oversizing proportion between the no THV displacement population and the THV displacement population: 90.6% vs. 75.0%). The added Dacron patches not only provide extra radial support for the AV but also increase the friction, which is conducive to anchoring. Furthermore, appropriate coaxiality and THV size may avoid postprocedural PVL occurrence and improve the hemodynamics [28]. Good coaxiality and the use of larger self-expandable valves can reduce the incidence of paravalvular aortic regurgitation [19]. Correspondingly, similar outcomes were obtained in our study (coaxiality: OR: 0.58, 95% CI: 0.21–0.72, *p* < 0.001; extra oversizing: OR: 0.66, 95% CI: 0.25–0.90, *p* < 0.001).

However, routine imaging assessments have not yet been able to meet the needs of accurate selection of the THV size and determination of the coaxial angle. Three-dimensional printing has been widely used in the cardiovascular field [18]. When it is performed before a procedure, 3D printing can be used to measure and simulate, which is conducive to evaluating and formulating a surgical plan [29]. When implanting the J-Valve system, it is very important to ensure that the projection direction is tangential to the annulus, and the implanted THV should be perpendicular to the annular plane and parallel to the long axis of the aorta. After the positioning key enters the sinuses, a repeated DSA must be carried out to confirm the morphology of the positioning keys. This step also ensures the coaxiality of the THV. When the THV is released to the annulus plane, special attention should be paid to whether the prosthesis is affected by the positioning keys. If the deformation caused by the positioning keys is ignored, the coaxiality and implanted morphology will be seriously influenced. Although surgeons should be fully familiar with the procedures, they may also use the pulsatile simulator to correctly perform the procedure after the occurrence of unexpected conditions for response and remedy, in order to improve the procedural success rate. The results of this study confirmed that preprocedural simulations may help surgeons accurately use extra oversizing (97.6% vs. 85.4%) and adjust the coaxiality angle (9.7 ± 3.9° vs. 12.7 ± 3.8°), thereby reducing the incidence of THV displacement (0 vs. 9.8%) and PVL (0 vs. 9.8%).

## 5. Study Limitations

This study has some limitations. First, the observational nature of this study will not avoid selection bias. Second, this is a multicenter study, and centralized echocardiography and CTA evaluations were lacking during the study. In addition, the extra oversizing and coaxial angle measurements used in this study were determined by the surgeons and engineers at each center, so there may be some deviations in the results. Fourth, as a result of the continuous development of 3D printing and materials science, the properties of the models were relatively close to those of the real anatomy or the aortic roots, but there are still large gaps that need to be improved and solved.

## 6. Conclusions

This is the first clinical study to evaluate the use of the J-Valve for transapical TAVR in the treatment of patients with noncalcified AR with a large annulus. We evaluated the safety and efficacy of the extra oversizing technique in such patients and concluded that extra oversizing and the coaxial angle were predictors of postprocedural THV displacement and ≥ mild PVL, thus demonstrating the reliability and feasibility of preprocedural 3D printing simulations.

## Availability of Data and Materials

The original contributions presented in the study are included in the article and in the supplementary material. Further inquiries can be directed to the corresponding author.

## Abbreviations

AR, aortic regurgitation; AS, aortic stenosis; AV, aortic valve; CTA, computed tomography angiography; LVEF, left ventricular ejection fraction; NYHA, New York Heart Association; PVL, paravalvular leakage; SAVR, surgical aortic valve replacement; STS, Society of Thoracic Surgeons; TAVR, transcatheter aortic valve replacement; TEE, transesophageal echocardiography; THV, transcatheter heart valve; TTE, transthoracic echocardiography.

## Supplementary Material

Supplementary material associated with this article can be found, in the online version, at https://doi.org/10.31083/j.rcm2509319.
