# Supplementary material for: Transapical Transcatheter Aortic Valve Replacement Under 3-Dimensional Guidance to Treat Pure Aortic Regurgitation in Patients with a Large Aortic Annulus
Source: Rev Cardiovasc Med. 2024 Sep 9;25(9):319. doi: 10.31083/j.rcm2509319 (PMC11440419; doi:10.31083/j.rcm2509319)
Supplement: Supplementary file 1 [file 2153-8174-25-9-319-s1.docx]

**Supplemental Data**

**
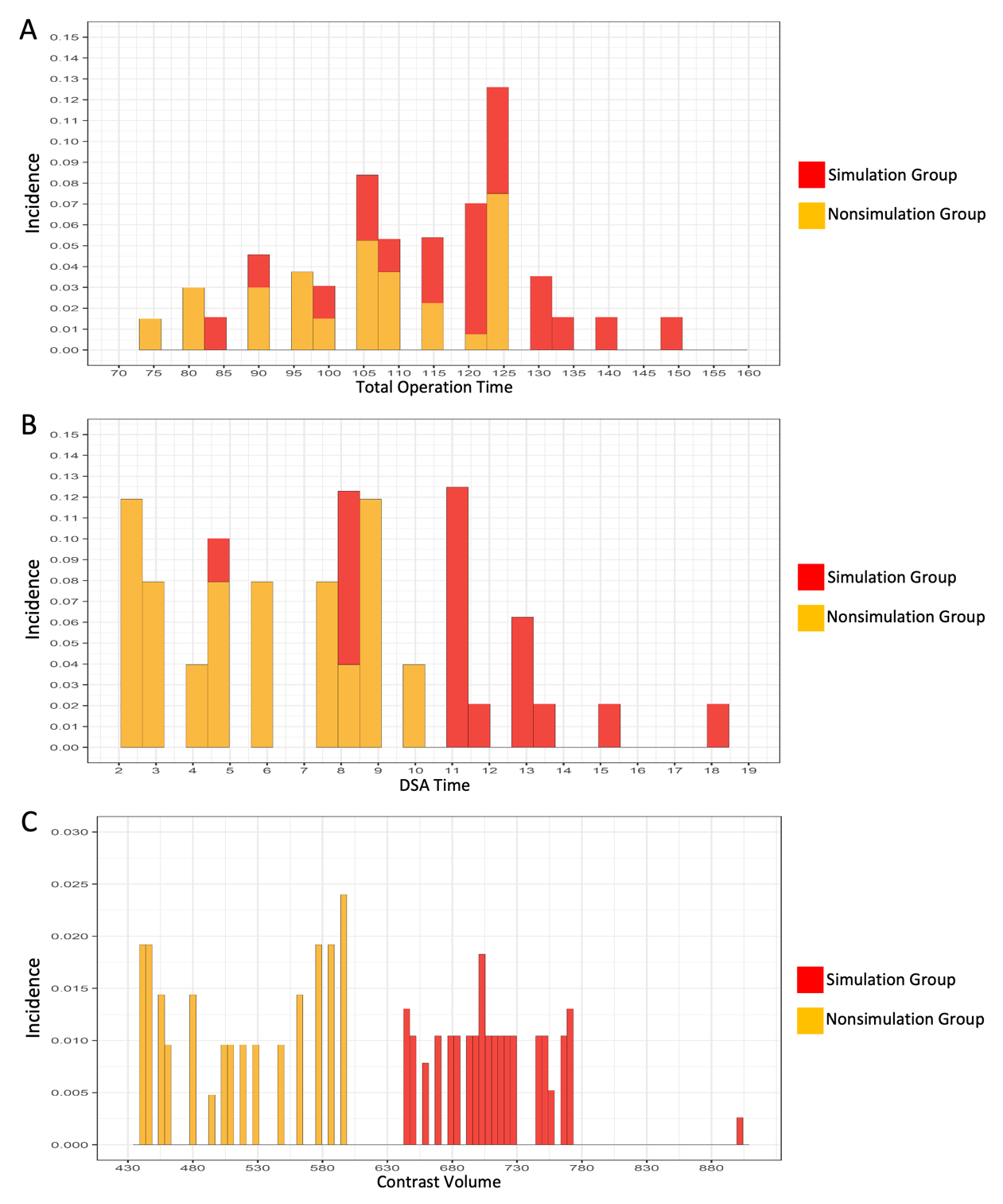
**

**Supplementary Fig. 1.** The total operating time, digital subtraction angiography time, and radiation amounts compared between the two groups.
